# Practice size, caseload, deprivation and quality of care of patients with coronary heart disease, hypertension and stroke in primary care: national cross-sectional study

**DOI:** 10.1186/1472-6963-7-96

**Published:** 2007-06-27

**Authors:** Sonia Saxena, Josip Car, Darren Eldred, Michael Soljak, Azeem Majeed

**Affiliations:** 1 Department of Primary Care and Social Medicine, Imperial College London, London W6 8RP, UK; 2 North West London Strategic Health Authority, London UK

## Abstract

**Background:**

Reports of higher quality care by higher-volume secondary care providers have fuelled a shift of services from smaller provider units to larger hospitals and units. In the United Kingdom, most patients are managed in primary care. Hence if larger practices provide better quality of care; this would have important implications for the future organization of primary care services. We examined the association between quality of primary care for cardiovascular disease achieved by general practices in England and Scotland by general practice caseload, practice size and area based deprivation measures, using data from the New General Practitioner (GP) Contract.

**Methods:**

We analyzed data from 8,970 general practices with a total registered population of 55,522,778 patients in England and Scotland. We measured practice performance against 26 cardiovascular disease (coronary heart disease, left ventricular disease, and stroke) Quality and Outcomes Framework (QOF) indicators for patients on cardiovascular disease registers and linked this with data on practice characteristics and census data.

**Results:**

Despite wide variations in practice list sizes and deprivation, the prevalence of was remarkably consistent, (coronary heart disease, left ventricular dysfunction, hypertension and cerebrovascular disease was 3.7%; 0.45%; 11.4% and 1.5% respectively). Achievement in quality of care for cardiovascular disease, as measured by QOF, was consistently high regardless of caseload or size with a few notable exceptions: practices with larger list sizes, higher cardiovascular disease caseloads and those in affluent areas had higher achievement of indicators requiring referral for further investigation. For example, small practices achieved lower scores 71.4% than large practices 88.6% (P < 0.0001) for referral for exercise testing and specialist assessment of patients with newly diagnosed angina.

**Conclusion:**

The volume-outcome relationship found in hospital settings is not seen between practices in the UK in management of cardiovascular disorders in primary care. Further work is warranted to explain apparently poorer quality achievement in some aspects of cardiovascular management relating to initial diagnosis and management among practices in deprived areas, smaller practices and those with a smaller caseload.

## Background

Prompted by numerous studies describing an inverse relationship between volume and outcome, several policy efforts are attempting to concentrate complex procedures in high-volume hospitals[1,2]. A systematic review of the volume-outcome relationship found that 71% of all studies of hospital volume and 69% of studies of physician volume reported a statistically significant association between higher volume and better health outcomes[1]. Reports of higher quality care by higher-volume providers are thus used by healthcare purchasers as one reason to shift services to larger hospitals and amalgamate smaller provider units into larger units [3-5].

Despite the considerable body of research on volume-outcome relationship in hospital and specialist settings, there is a dearth of research on volume-outcome relationship in primary care setting. One recent study reported that smaller practices could provide better access to a general practitioner-GP but that quality of diabetes care was better in larger practices[6]. Since most patients are managed in primary care, if larger practices provide better quality of care, this would have important implications for the future organization of primary care services.

In April 2004, a new contract was introduced for general practitioners (the sole type of primary care physician in the United Kingdom) in which a significant proportion of general practice income would come from performance against targets in a new Quality and Outcomes Framework[7]. The new contract represents a major innovation in the organization of primary care services and the first time that pay for performance has been used on this scale in any health care system. It provides standardized information on the quality of care in general practices nationally and unique data to measure the quality of primary care experienced by the entire national population. In the first year following implementation English general practices attained a uniformly high level of achievement of this new pay-for-performance contract[8].

To address the question whether higher volume of cases or larger practice size leads to improved quality of care, we examined the achievement of process of care and intermediate outcomes for people with cardiovascular diseases (coronary heart disease, left ventricular dysfunction, hypertension and cerebrovascular disease) in English and Scottish general practice by general practice caseload, practice size and area based deprivation measures, using data from the New General Practitioner Contract[7].

## Methods

### Data sources

The study was carried out using Quality and Outcomes Framework (QOF) data for England and Scotland collected from April 2004 to March 2005[8]. The data comprise 9,411 general practices with a population of 57,787,662 patients. Since data are anonymised the study was exempt from the need for ethical approval. Practices score points based on their levels of achievement against a range of evidence-based clinical indicators in 10 chronic disease areas including cardiovascular disease, which accounts for 47% of the total clinical points, as well as points in practice organization and management. Payments to practice are calculated from points achieved.

There are two types of data: data relating to QOF indicator or domain scores and disease prevalence information for each disease within the clinical domain of the QOF. The source of QOF data is a national IT system called the Quality Management and Analysis System (QMAS)[9]. This single national system ensures consistency in the calculation of quality achievement and prevalence. Clinical QOF data is extracted from individual general practice clinical systems and sent automatically to QMAS; organizational, access, patient experience and additional service indicators are entered by the practice directly into QMAS via a web-browser. Data from practices without QMAS-compliant computer systems were entered manually into QMAS (exceptionally few practices do not have QMAS-compliant computer systems). QOF information is collected at an aggregate level for each general practice and there is no patient-specific data within QMAS.

Core funding of English and Scottish general practices is based on the number of patients, adjusted for characteristics of the patients and the area, including area based deprivation. QOF payments represent additional incentive earning for the practices. For the clinical indicators, practices receive points that generate payments according to the proportion of patients for whom they achieve each target. Points are awarded on a sliding scale within the payment range with the minimum 25% and maximum varying between 50–90% (Table 1). For example, to receive any payment for coronary heart disease indicator number 8 practices had to achieve for at least 25% of their patients target total cholesterol 5 mmol/l or less – measured in the last 15 months; and to be receive maximum number of points at least 60% of patients had to achieve that target cholesterol level. Practices were paid £77.50 ($135) per point, adjusted for the relative prevalence of the disease (payment is multiplied by the square root of the prevalence of the disease among the patients served by the practice and divided by the square root of the mean national prevalence of the disease). Payment is further adjusted for list size relative to national list size.

**Table 1:** Twenty-six Quality indicators for management of cardiovascular diseases.

**Secondary Prevention in Coronary Heart Disease **(Description of Indicators)	Points*	Target**
CHD 2. The percentage of patients with newly diagnosed angina (diagnosed after 01/04/03) who are referred for exercise testing and/or specialist assessment	7	90%
CHD 3. The percentage of patients with coronary heart disease, whose notes record smoking status in the past 15 months, except those who have never smoked where smoking status need be recorded only once	7	90%
CHD 4. The percentage of patients with coronary heart disease who smoke, whose notes contain a record that smoking cessation advice has been offered within the last 15 months	4	90%
CHD 5. The percentage of patients with coronary heart disease whose notes have a record of blood pressure in the previous 15 months	7	90%
CHD 6. The percentage of patients with coronary heart disease, in whom the last blood pressure reading (measured in the last 15 months) is 150/90 or less	19	70%
CHD 7. The percentage of patients with coronary heart disease whose notes have a record of total cholesterol in the previous 15 months	7	90%
CHD 8. The percentage of patients with coronary heart disease whose last measured total cholesterol (measured in the last 15 months) is 5 mmol/l or less	16	60%
CHD 9. The percentage of patients with coronary heart disease with a record in the last 15 months that aspirin, an alternative anti-platelet therapy, or an anti-coagulant is being taken (unless a contraindication or side effects are recorded)	7	90%
CHD 10. The percentage of patients with coronary heart disease who are currently treated with a beta blocker (unless a contraindication or side-effects are recorded)	7	50%
CHD 11. The percentage of patients with a history of myocardial infarction (diagnosed after 1 April 2003) who are currently treated with an ACE inhibitor	7	70%
CHD 12. The percentage of patients with coronary heart disease who have a record of influenza vaccination in the preceding 1 September to 31 March	7	85%

**Subset – Left Ventricular Dysfunction**		

LVD 2. The percentage of patients with a diagnosis of CHD and left ventricular dysfunction (diagnosed after 1/4/03) which has been confirmed by an echocardiogram	6	90%
LVD 3. The percentage of patients with a diagnosis of CHD and left ventricular dysfunction who are currently treated with ACE inhibitors (or A2 antagonists)	10	70%

**Stroke or transient ischemic attacks**		

CVA 2. The percentage of new patients with presumptive stroke (presenting after 01/04/03) who have been referred for confirmation of the diagnosis by CT or MRI scan	2	80%
CVA 3. The percentage of patients with TIA or stroke who have a record of smoking status in the last 15 months, except those who have never smoked where smoking status should be recorded at least once since diagnosis	3	90%
CVA 4. The percentage of patients with a history of TIA or stroke who smoke and whose notes contain a record that smoking cessation advice has been offered in the last 15 months	2	70%
CVA 5. The percentage of patients with TIA or stroke who have a record of blood pressure in the notes in the preceding 15 months	2	90%
CVA 6. The percentage of patients with a history of TIA or stroke in whom the last blood pressure reading (measured in the last 15 months) is 150/90 or less	5	70%
CVA 7. The percentage of patients with TIA or stroke who have a record of total cholesterol in the last 15 months	2	90%
CVA 8. The percentage of patients with TIA or stroke whose last measured total cholesterol (measured in the last 15 months) is 5 mmol/l or less	5	60%
CVA 9. The percentage of patients with a stroke shown to be nonhemorrhagic, or a history of TIA, who have a record that aspirin, an alternative anti-platelet therapy, or an anti-coagulant is being taken (unless a contraindication or side-effects are recorded)	4	90%
CVA 10. The percentage of patients with TIA or stroke who have had influenza immunization in the preceding 1 September to 31 March	2	85%

**Hypertension**		

BP 2. The percentage of patients with hypertension whose notes record smoking status at least once	10	90%
BP 3. The percentage of patients with hypertension who smoke, whose notes contain a record that smoking cessation advice has been offered at least once	10	90%
BP 4. The percentage of patients with hypertension in which there is a record of the blood pressure in the past 9 months	20	90%
BP 5. The percentage of patients with hypertension in whom the last blood pressure (measured in last 9 months) is 150/90 or less	56	70%

*For each indicator practice could achieve from 0 up to 56 points – as specified for each indicator. **Percentage of patients that needed to be reviewed to receive maximum points for an indicator (as specified in the points column). All minimum threshold targets for payment are 25%.

Abbreviations: BP – Hypertension, CHD – Coronary Heart Disease, LVD – Left Ventricular Dysfunction, CVA – Cerebral Vascular Accident;

### Linking deprivation measures to practices

We obtained Index of Multiple Deprivation (IMD) 2004 data at super output area (SOA) level for England from the Department of Transport, Local Government and the Regions [Table 2]. The IMD 2004 is commonly used for measuring area based deprivation in England and includes 37 indicators under seven domains: income deprivation; employment deprivation; health deprivation and disability; education, skills and training deprivation; living environment deprivation; barriers to housing and services; and crime. There are 32,482 SOAs in England, each with a minimum of 1000 residents and 400 households. They are part of a new geographical hierarchy designed to improve the statistical comparisons that are made between small areas. Each SOA is assigned an IMD score, with high scores implying higher levels of deprivation. For this study, we were able to link practice identifying codes and postcode (i.e. zipcode) were linked with the Quality and Outcome Framework data using established reference tables[10,11]. Practices were mapped to postcode locations using GIS software MapInfo Professional 7.8[12] and linked to area based deprivation scores for England[13] and Scotland[14]. Differences between area based deprivation scores for England and Scotland are small and thus combined analysis of data was possible[15].

**Table 2:** Practice characteristics and prevalence of cardiovascular disorders.

**List size**	**No. of practices**	**Percent of practices**	**Percent of patients**	**Average list size**	**CHD prevalence**	**LVD prevalence**	**Hypertension prevalence**	**CVA prevalence**
0 to 2,999	2058	22.9%	8.1%	2,192	3.6%	0.4%	10.9%	1.5%
3,000 to 4,999	2081	23.2%	14.7%	3,924	3.6%	0.4%	11.4%	1.5%
5,000 to 7,999	2305	25.7%	26.6%	6,402	3.7%	0.5%	11.7%	1.6%
8,000 to 9,999	1101	12.3%	17.8%	8,954	3.8%	0.5%	11.6%	1.6%
> 9,999	1425	15.9%	32.8%	12,793	3.6%	0.4%	11.3%	1.5%
**Deprivation score^# ^– Size**								
1 – 0 to 2,999	1,012	11.3%	4.1%	2,240	3.5%	0.4%	10.2%	1.1%
1 – 3,000 to 4,999	887	9.9%	6.2%	3,889	3.5%	0.4%	10.5%	1.2%
1 – 5,000 to 7,999	887	9.9%	10.2%	6,365	3.7%	0.5%	11.0%	1.4%
1 – 8,000 to 9,999	387	4.3%	6.3%	8,976	4.0%	0.5%	11.4%	1.7%
1 – 10,000 or Over	462	5.2%	10.4%	12,545	3.9%	0.5%	11.3%	1.6%
2 – 0 to 2,999	736	8.2%	2.8%	2,120	3.8%	0.5%	11.7%	1.4%
2 – 3,000 to 4,999	745	8.3%	5.3%	3,941	3.8%	0.5%	11.8%	1.5%
2 – 5,000 to 7,999	843	9.4%	9.7%	6,415	3.9%	0.5%	11.8%	1.6%
2 – 8,000 to 9,999	413	4.6%	6.6%	8,917	3.9%	0.5%	11.8%	1.7%
2 – 10,000 or Over	538	6.0%	12.5%	12,922	3.7%	0.5%	11.4%	1.6%
3 – 0 to 2,999	310	3.5%	1.2%	2,204	3.4%	0.4%	11.4%	1.3%
3 – 3,000 to 4,999	448	5.0%	3.2%	3,964	3.3%	0.4%	11.7%	1.4%
3 – 5,000 to 7,999	575	6.4%	6.7%	6,442	3.5%	0.4%	11.8%	1.5%
3 – 8,000 to 9,999	301	3.4%	4.9%	8,976	3.5%	0.4%	11.4%	1.6%
3 – 10,000 or Over	425	4.7%	9.9%	12,900	3.2%	0.4%	11.1%	1.5%

See Table 1 for explanation of abbreviation

^#^Practices are grouped into three bands according to their deprivation scores 1 deprived 2 intermediate 3 affluent based on area based measures. ^[13,14]^

### Exclusions and exception reporting

Practices were excluded if they could not be matched via their postcode. We excluded practices in Scotland that were not fully part of the contract. In total, we excluded 441 (4.1%) practices with 2,264,884 (3.5%) patients, leaving 8,970 general practices with a total list size of 55,522,578 for analysis. From the Quality and Outcomes Framework (QOF) data, we excluded indicators reporting the presence of a disease register since all practices in QOF met this requirement. We used Stata version 9 for all analyses[16].

The Quality and Outcomes Framework allows exception reporting to avoid practices being penalized unfairly. For example, where patients do not attend for review, or if a medication cannot be prescribed due to a contraindication or side-effect. The criteria for exception reporting include: patients who are on maximum tolerated doses of medication whose levels remain sub-optimal; where a patient does not agree to investigation or treatment (informed dissent), and this has been recorded in their medical records; and where an investigative service or secondary care service is unavailable. Level of exception reporting was monitored for each individual practice and if it would be unusually high or low the data would need to be verified. The median rate of exception reporting after the first year was small <6% and we did not make any adjustment for this[8]. Primary care organizations are responsible for monitoring exception reporting in practices.

### Analysis

We grouped practices into quintiles of list size; five groups based on the number of patients registered with the practice. We grouped practices into quintiles of prevalence, five groups based on the number of patients with cardiovascular disorders (coronary heart disease, hypertension and stroke) in each practice. Groupings of practices into three categories (tertiles): 1-deprived 2-intermediate and 3-affluent, based on the national rank of the geographic area in which the practice is located (i.e. practices in group 1 represent 33.3% of neighborhoods with lowest socio-economic status nationally). We calculated the percentage of practices who achieved each of the targets by caseload, practice size and deprivation.

## Results

In total, there were 2,039,919 people with coronary heart disease and 250,925 people with coronary heart disease and left ventricular dysfunction in the 8,970 general practices in this study. Prevalence of coronary heart disease was 3.67% and prevalence of coronary heart disease and left ventricular dysfunction 0.45%. Prevalence of hypertension was 11.35% (6,300,476 patients) and 1.51% for cerebrovascular disease (839,758 patients).

### Practice characteristics and cardiovascular disorders

Practice size varied widely from 52 to 36,130 patients (mean 6,189). The number of cases in individual practices varied from 0 to 1,994 (mean 227) for coronary heart disease and the prevalence of coronary heart disease varied from 0% to 34.61% (mean 3.67%). Scores achieved for recording and management of twenty six indicators among patients with cardiovascular disorders are shown in Tables 3, 4, 5. Despite this variation the prevalence of cardiovascular disorders reported through the QOF was consistent for practices regardless of practice list size.

**Table 3:** Inter-practice variation in quality of care scores for coronary heart disease and left ventricular dysfunction according to caseload.

**CHD caseload**	CHD2*	CHD3*	CHD4*	CHD5*	CHD6*	CHD7*	CHD8*	CHD9*	CHD10*	CHD11*	CHD12*	LVD caseload*	LVD2*	LVD3*
1: Less than 87	71.4	94.3	91.8	95.3	84.2	87.2	68.3	88.5	65.8	87.6	86.0	1: Less than 8	73.3	86.6
2: 87 to 147	78.3	95.0	92.6	96.2	84.1	89.2	69.8	89.1	64.3	88.1	86.8	2: 8 to 16	80.5	84.1
3: 148 to 232	83.0	95.2	93.0	96.5	84.1	90.2	71.7	89.8	65.3	87.2	87.1	3: 17 to 27	85.3	83.4
4: 233 to 352	86.6	95.2	93.1	96.6	84.6	90.5	72.8	90.5	65.8	86.9	87.4	4: 28 to 44	87.3	82.9
5: 353 or More	88.6	95.1	93.1	96.6	84.1	90.4	72.5	90.5	65.2	86.3	87.2	5: 45 or More	89.3	81.5

See Table 1 for explanation of abbreviation

*Percentage achievement in 5 groups significantly different using Kruskal Wallis exact test (P < 0.0001)

**Table 4:** Inter-practice variation in quality of care scores for hypertension and stroke according to caseload.

**BP Caseload**	BP2*	BP3*	BP4*	BP5*	**CVA caseload**	CVA2*	CVA3*	CVA4*	CVA5*	CVA6*	CVA7*	CVA8*	CVA9*	CVA10*
1: Less than 281	92.6	92.0	88.5	71.0	1: Less than 28	63.4	92.6	88.5	93.6	81.3	80.8	59.7	88.1	82.9
2: 281 to 477	94.0	93.5	89.9	72.1	2: 28 to 54	75.6	93.4	91.0	94.7	81.3	83.6	62.1	88.8	83.9
3: 478 to 728	94.4	94.3	90.1	71.7	3: 55 to 95	80.7	93.6	90.4	95.1	81.8	85.2	63.7	89.6	84.5
4: 729 to 1070	94.4	94.2	90.3	71.5	4: 96 to 149	84.9	93.2	89.8	95.2	81.7	85.2	64.1	90.0	84.5
5: 1071 or More	94.4	94.6	90.3	71.2	5: 150 or More	86.0	92.6	89.4	95.1	81.3	84.4	63.1	89.5	84.4

See Table 1 for explanation of abbreviations

*Percentage achievement in 5 groups significantly different using Kruskal Wallis exact test (P < 0.0001)

**Table 5:** Inter-practice variation in quality of care scores for coronary heart disease and left ventricular dysfunction by practice size and deprivation

**Deprivation score^# ^– Size**	CHD2*	CHD3*	CHD4*	CHD5*	CHD6*	CHD7*	CHD8*	CHD9*	CHD10*	CHD11*	CHD12*	LVD2*	LVD3*
1 – 0 to 2,999	66.7	93.7	90.3	94.7	82.5	86.2	66.2	87.5	62.9	87.2	84.8	70.9	84.4
1 – 3,000 to 4,999	76.0	94.9	92.5	95.8	82.8	88.7	69.3	89.1	63.9	87.8	85.9	76.8	83.6
1 – 5,000 to 7,999	82.6	94.6	92.4	96.0	82.8	89.0	70.1	89.3	64.3	87.5	85.6	82.9	82.7
1 – 8,000 to 9,999	85.6	94.5	91.9	96.4	83.3	90.0	71.8	89.9	64.1	86.3	85.8	86.5	82.1
1 – 10,000 or Over	87.2	94.9	92.7	96.5	83.7	90.2	71.8	90.1	64.6	86.2	86.5	87.2	81.5
2 – 0 to 2,999	72.4	94.2	91.4	95.6	84.6	87.3	68.0	88.5	66.1	86.1	86.6	74.8	83.4
2 – 3,000 to 4,999	81.7	95.5	93.4	96.2	85.6	90.1	71.6	89.9	66.1	87.5	87.6	84.1	84.1
2 – 5,000 to 7,999	85.5	95.4	92.9	96.6	84.8	90.5	72.6	90.3	66.1	87.4	87.5	87.8	83.3
2 – 8,000 to 9,999	88.4	95.4	94.2	96.7	84.7	90.3	73.0	90.7	66.6	86.4	87.2	89.8	83.8
2 – 10,000 or Over	91.0	95.2	93.5	96.7	84.8	91.0	73.3	90.7	66.2	86.3	87.5	91.7	82.8
3 – 0 to 2,999	77.5	95.2	93.3	96.5	85.3	90.0	70.1	88.9	65.9	87.8	89.2	82.5	84.4
3 – 3,000 to 4,999	84.2	95.4	94.2	97.2	85.6	91.3	73.2	90.3	67.1	88.3	88.7	85.7	86.0
3 – 5,000 to 7,999	88.8	95.7	93.9	97.0	85.5	91.3	74.5	91.1	66.7	87.5	88.5	90.5	84.5
3 – 8,000 to 9,999	90.9	95.8	94.0	97.1	85.7	91.5	74.1	91.4	66.4	87.6	88.8	93.1	83.6
3 – 10,000 or Over	91.2	95.6	94.6	97.0	85.1	91.2	74.4	91.2	65.8	87.6	88.2	92.5	83.5

See Table 1 for explanation of abbreviations

^#^Practices are grouped into three bands according to their deprivation scores 1 deprived 2 intermediate 3 affluent based on area based measures ^[13,14]^

*Percentage achievement in 15 groups significantly different using Kruskal Wallis exact test (P < 0.0001)

### Caseload, practice size and quality of care

For most of the indicators there was a high level of quality achievement regardless of caseload, except for selected indicators for early diagnostic investigations, where practices with a higher caseload of patients with cardiovascular had a higher level of achievement of indicators of quality (Table 3). Practices with lowest caseload achieved the score of 71.4% for referral of patients with newly diagnosed angina (diagnosed after 1/4/03) for exercise testing and/or specialist assessment (CHD 2) whereas the practices with highest caseload achieved 88.6%. Similarly, practices with low caseload achieved 73.3% score (the points achieved as a percentage of points available) for the percentage of patients with a diagnosis of coronary heart disease and left ventricular dysfunction which has been confirmed by an echocardiogram (LVD2), whereas the practices with highest caseload achieved 89.3%. Similarly, low caseload practices achieved 63% of referral for confirmation of newly diagnosed presumptive stroke by CT or MRI scan compared with 86% of practices with the highest caseloads (Table 4).

### Deprivation and quality of care

The prevalence of all the cardiovascular disorders studies was remarkably consistent between strata of area based deprivation and list size. The effect of the caseload and practice size described above was increased when practices were compared in addition to their caseload/list size also according to the practice area based deprivation (Table 5 and 6). The association between practice area based deprivation and quality of care was strongest for patients with newly diagnosed angina who are referred for exercise testing and/or specialist assessment. For most other indicators of cardiovascular disorder management, there were weak associations with area based deprivation (Tables 5 and 6).

**Table 6:** Inter-practice variation in quality of care scores for hypertension and stroke according to practice size and area based deprivation.

**Deprivation score^# ^Size**	BP2*	BP3*	BP4*	BP5*	CVA2*	CVA3*	CVA4*	CVA5*	CVA6*	CVA7*	CVA8*	CVA9*	CVA10*
1 – 0 to 2,999	92.6	91.9	88.3	70.3	63.8	91.9	88.5	93.4	80.1	81.0	59.9	87.8	82.1
1 – 3,000 to 4,999	93.6	93.5	89.0	70.1	71.3	92.9	89.3	94.1	79.6	83.1	61.8	89.0	83.1
1 – 5,000 to 7,999	93.6	93.6	89.4	69.8	78.2	92.6	88.8	94.4	79.9	82.8	62.3	88.7	82.2
1 – 8,000 to 9,999	93.9	93.8	89.6	69.6	82.3	91.8	88.1	94.3	79.8	82.6	62.2	88.4	81.3
1 – 10,000 or Over	93.8	94.2	89.8	70.2	84.0	92.2	88.9	94.7	80.2	83.1	61.7	88.5	82.4
2 – 0 to 2,999	93.9	93.1	89.2	72.4	69.3	93.1	90.7	94.7	82.3	82.5	60.6	88.7	84.7
2 – 3,000 to 4,999	94.5	93.9	90.1	73.4	80.2	94.0	91.0	95.2	83.2	85.3	64.1	89.3	85.1
2 – 5,000 to 7,999	94.5	94.3	90.3	71.9	82.9	93.3	89.6	95.0	81.9	84.9	63.5	90.0	84.5
2 – 8,000 to 9,999	94.6	94.6	90.5	71.6	85.0	93.3	90.0	95.2	82.0	84.9	63.8	90.2	84.1
2 – 10,000 or Over	94.2	94.7	90.2	71.7	87.0	92.9	90.0	95.2	82.1	84.5	63.0	90.0	84.3
3 – 0 to 2,999	94.1	93.3	90.9	74.0	76.8	93.8	90.5	94.6	82.6	84.6	62.7	88.8	87.0
3 – 3,000 to 4,999	94.2	93.9	91.0	73.0	82.0	94.1	91.5	95.9	83.3	85.9	63.7	89.9	86.3
3 – 5,000 to 7,999	94.7	94.1	91.0	73.0	85.8	94.3	91.7	96.1	83.6	86.5	65.5	90.5	86.2
3 – 8,000 to 9,999	94.6	94.9	90.7	72.5	87.3	93.6	90.8	95.4	82.8	86.1	64.2	90.2	85.4
3 – 10,000 or Over	94.2	94.9	90.4	72.0	87.3	93.7	91.2	95.3	82.3	84.8	63.2	89.9	85.6

See Table 1 for explanation of abbreviations.

^#^Practices are grouped into three bands according to their deprivation scores 1 deprived 2 intermediate 3 affluent based on area based measures (Index of multiple deprivation-IMD), Office of the Deputy Prime Minister. Indices of deprivation 2004. ^[13,14]^

*Percentage achievement in 15 groups significantly different using Kruskal Wallis exact test (P < 0.0001)

## Discussion

### Summary of main findings

Despite wide variations in practice size and deprivation levels, we found surprisingly little variation between general practices in the prevalence and management of cardiovascular disorders. Practices with a higher caseload had similar achievement of quality for many aspects of management, but practices with higher caseload achieved higher scores, and in larger practices and more affluent practices for some indicators for initial diagnosis and management including referrals for exercise testing and/or specialist assessment, an echocardiogram, CT or MRI scan. This may reflect better access to resources for initial management in larger practices than smaller ones, perhaps through local planning and commissioning of resources by primary care trusts for services where there is a higher caseload.

### Findings in relation to other studies

Two principal hypotheses have been used to explain volume-outcome relationships: 1) Physicians (and hospitals) develop more effective skills if they treat more patients ("practice makes perfect"); or 2) physicians (and hospitals) achieving better outcomes receive more referrals and thus accrue larger volumes ("selective referral")[1]. While the first hypothesis may particularly apply to our findings (e.g. more accurate case ascertainment), the latter is less likely to apply to NHS primary care than secondary care and since UK general practitioners usually provide all round general medical services rather than specialist chronic disease management and patients' ability to choose their GP is largely determined by whether they live within practice catchment areas. We cannot test either hypothesis with our current data. Moreover, we believe that other explanations may be more plausible in primary care volume-outcome relationships; principally, how the delivery of care is organized and shared between the members of the primary care team. This is supported by one study reporting that organizational domains in the new contract were associated with lower achievement in smaller practices compared with larger ones, whereas scores for clinical care were similar[17]. Chronic disease management can be complex and require input from many different elements of the primary care team. Many of the processes of care assessed in our study could be effectively performed by nurses, for example checking of blood cholesterol. Such sharing of care may be more easily achievable in larger practices, which can generally employ a wider range of staff. Indeed, it is known that many practices employed additional nurses and other supporting staff when the new contract was introduced.

A recent study suggests that the "wealth-health gradient" in cardiovascular mortality may be partially ameliorated by more rigorous management of known risk factors among less affluent people[18]. Our findings that prevalence of cardiovascular disorders was similar in deprived and affluent practices is unexpected and, in this light, our finding that some aspects of the management of cardiovascular diseases were worse in practices in deprived areas is of concern and consistent with one other study reporting that socially deprived areas experience a lower quality of care overall[19]. However, most aspects of risk management showed relatively little association with practice area based deprivation. This suggests that people living in neighborhoods with low socio-economic status are now receiving cardiovascular disease management in primary care of similar quality to people living in more affluent neighborhoods.

### Strengths and limitations

This is the largest population-based study to date examining volume-outcome association for cardiovascular disorders in primary care. The structure of primary care in the United Kingdom offers some unique opportunities for examining the association between volume and outcome. Almost the entire population is registered with a general practitioner, who is responsible for providing primary care services and arranging referrals for specialist care. This means that general practitioners have well-defined denominator populations, which in turn allows the calculation of accurate disease prevalence and treatment rates.

One limitation of the study is that patients with cardiovascular disorders but not coded on the computer record would not have been identified. However, general practices are now paid by the results they achieve and also on prevalence. Quality and Outcomes Framework data also have some other limitations. There is no information on gender, age, ethnicity and severity of disease or co morbidity or individual patients' socio-economic status (this was assessed indirectly through practice area based deprivation scores and is not necessarily a good marker of deprivation for individual registered patients) and thus no adjustment for these is possible. However, even if there were systematic difference in patient characteristics and case-mix, practices still had the option to 'exception report' patients, thus enabling the practices to still achieve maximum scores. When patients are 'exception reported' they are excluded from that score. Initial reports suggest that only a small minority of practices achieved high scores by exception reporting[8]. There is also a risk of manipulation or gaming (for example, recording a patient's blood pressure as being lower than it actually is) which will be difficult to detect. Although this may occur in some areas, practices are subject to an annual detailed inspection and the penalties for making fraudulent claims are severe[7].

There was also limited information about practice characteristics. We defined practices with large list sizes as large practices but did not have information about the number of GPs employed by a practice. Hence the caseload of a condition per GP may be similar or lower to that in smaller practices even if the caseload per practice is high. We were unable to account for this since we did not have information about the number of GPs employed per practice. Other possible explanations for the differences we found in quality achievement for referring patients with newly diagnosed cardiovascular disorders to early diagnostic facilities, such as local waiting lists or urban-rural differences in access that we felt went beyond the scope of this study.

Although the indicators of the quality of care are 'evidence-based', they are process measures or intermediate outcomes. We have no data on outcomes such as mortality or quality of life. Our findings are also limited to the management of cardiovascular disorders and we cannot say whether similarly weak volume-quality relationships would occur in the management of other diseases in primary care. Finally, our study is unable to explain the mechanism through which volume may influence quality in primary care.

Although numerous studies have examined the volume-outcome relationship in secondary care,[20] very few previous studies have examined this relationship in primary care. Hippisley-Cox and colleagues compared a number of areas of practice activity in small and larger practices in the Trent region of England[21]. They found no strong evidence that small practices offered poorer quality of care than larger practices. Another study in London also found no great associations between practice size and quality of care[22].

## Conclusion

In contrast to the volume-outcome relationship found in hospital settings, we found that little relationship between the quality of care provided in management of cardiovascular disorders and practice caseload or practice size. This will have important implications for the organization of primary care services and suggests that merging practices into larger units may not have a major impact on the quality of clinical care offered for cardiovascular disease management in primary care. However, further work is warranted to explain apparently poorer quality achievement in some aspects of cardiovascular management relating to initial diagnosis and management among practices in deprived areas, smaller practices and those with a smaller caseload.

## Competing interests

The author(s) declare that they have no competing interests.

## Authors' contributions

AM and JC conceived the study. DE performed the statistical analyses. All authors contributed to the data interpretation. JC wrote the first draft of the manuscript and all authors contributed to the revision, read and approved the final manuscript. SS and AM are the guarantors for the study.

## Pre-publication history

The pre-publication history for this paper can be accessed here:


